# EuMoBot: replicating euglenoid movement in a soft robot

**DOI:** 10.1098/rsif.2018.0301

**Published:** 2018-11-21

**Authors:** Krishna Manaswi Digumarti, Andrew T. Conn, Jonathan Rossiter

**Affiliations:** 1 Bristol Robotics Laboratory, University of Bristol, Bristol BS16 1QY, UK; 2 Department of Mechanical Engineering, University of Bristol, Bristol BS8 1TR, UK; 3 Department of Engineering Mathematics, University of Bristol, Bristol BS8 1UB, UK

**Keywords:** soft robotics, bioinspired robotics, euglenoid movement, shape characterization, robot locomotion

## Abstract

Swimming is employed as a form of locomotion by many organisms in nature across a wide range of scales. Varied strategies of shape change are employed to achieve fluidic propulsion at different scales due to changes in hydrodynamics. In the case of microorganisms, the small mass, low Reynolds number and dominance of viscous forces in the medium, requires a change in shape that is non-invariant under time reversal to achieve movement. The *Euglena* family of unicellular flagellates evolved a characteristic type of locomotion called euglenoid movement to overcome this challenge, wherein the body undergoes a giant change in shape. It is believed that these large deformations enable the organism to move through viscous fluids and tiny spaces. The ability to drastically change the shape of the body is particularly attractive in robots designed to move through constrained spaces and cluttered environments such as through the human body for invasive medical procedures or through collapsed rubble in search of survivors. Inspired by the euglenoids, we present the design of EuMoBot, a multi-segment soft robot that replicates large body deformations to achieve locomotion. Two robots have been fabricated at different sizes operating with a constant internal volume, which exploit hyperelasticity of fluid-filled elastomeric chambers to replicate the motion of euglenoids. The smaller robot moves at a speed of 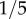 body lengths per cycle (20 mm min^−1^ or 2.2 cycles min^−1^) while the larger one attains a speed of 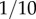 body lengths per cycle (4.5 mm min^−1^ or 0.4 cycles min^−1^). We show the potential for biomimetic soft robots employing shape change to both replicate biological motion and act as a tool for studying it. In addition, we present a quantitative method based on elliptic Fourier descriptors to characterize and compare the shape of the robot with that of its biological counterpart. Our results show a similarity in shape of 85% and indicate that this method can be applied to understand the evolution of shape in other nonlinear, dynamic soft robots where a model for the shape does not exist.

## 1. Introduction

Swimming as a form of locomotion is employed by many organisms in nature. From microscopic bacteria and algae to large organisms such as squids and whales, this method of locomotion is seen at a wide variety of scales. To swim, an organism needs to generate forwards movement through the fluid by generating propulsive forces intrinsically. This is typically achieved through cyclic changes in the shape of the body. The periodic movement of fins in fish, the rhythmic movement of arms and legs in a human, and the cyclic beating of cilia in a microorganism are some examples. There are however, differences in swimming strategies in organisms due to changes in hydrodynamics with scale.

At larger scales, a simple strategy exploiting inertial forces such as a forward and backward change in shape at different velocities is sufficient to generate movement, for example, in bivalve molluscs. In the case of smaller organisms like protozoa, locomotion through a fluid falls under a low Reynolds number regime. Owing to the domination of viscous forces over inertial forces in such a scenario, swimming requires a non-reciprocal change in shape to achieve propulsion [1].

**Figure 1.**
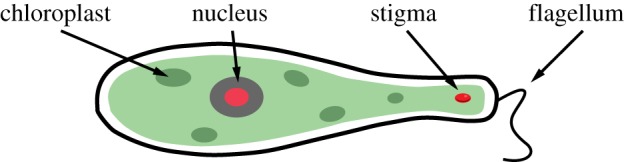
Schematic of a euglenoid organism showing various cellular organelles including a flagellum. Image adapted from [7]. Copyright © IEEE, 2017. (Online version in colour.)

**Figure 2.**
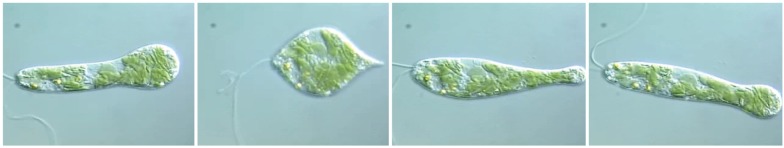
Sequence of images from video recordings of *Eutreptiella spirogyra* during euglenoid movement. Pictures reproduced with permission from Richard E. Triemer, The Euglenoid Project [8]. (Online version in colour.)

Euglenoids are unicellular flagellates that employ a unique strategy to swim at low Reynolds numbers. These organisms have been extensively studied in the laboratory as models because they demonstrate both plant-like and animal-like characteristics [2]. While the taxonomy and ecology of these cells has been studied comprehensively, studies on their locomotion are relatively recent [3–6]. Cells of euglenoids are typically equipped with one or more flagella (figure 1). Depending on the environment, they may use the flagella to swim or may exhibit a slower characteristic type of locomotion called euglenoid movement (figure 2), in which the cell undergoes a drastic change in shape. It is the second form of locomotion that is addressed in this study and is the inspiration for the design of our soft robot. Several researchers have taken inspiration from the animal kingdom to design robots that move in interesting ways. These are usually based on larger animals and are typically built to or at a smaller scale. Our approach is novel in that it is based on the behaviour of a microorganism and as shall be shown, can be used to design a functional robot at a larger scale.

Many organisms in nature possess the ability to change shape and deform their bodies. Deformations in vertebrates are usually constrained by rigid skeletal structures and limits of joints. On the other hand, invertebrates such as cephalopods, are able to squeeze through extremely small holes and manipulate objects of various shapes and sizes. Smaller organisms such as bacteria and algae use body deformations for feeding and to overcome challenges in their environments. Euglenoids stand out as unique among microorganisms because of the giant changes in body shape that they exhibit.

The reason for the evolution of euglenoid movement is not known. It is believed that deformations in the cell arise as a reaction to a strong stimulus such as light, heat, chemical shock or contact with an object [2]. When euglenoids were placed in a fluid-filled microscopic maze that closely resembles their natural environment [9], a large portion of them demonstrated euglenoid movement. This suggests that their characteristic locomotion is well suited for movement through a constrained environment. Such an ability would be particularly useful in a robot when traversing cluttered environments and squeezing through tight spaces. Examples of such scenarios include robots operating within the confines of the human body for invasive medical procedures, those looking for survivors in collapsed rubble and those evaluating the integrity of pipelines. The field of soft robotics has the potential to capture these large-body deformations in a robotic form.

The focus of this paper is (i) the replication of euglenoid-like locomotion in a soft robot and (ii) the use of a mathematical tool to quantify its motion and compare it to that of the euglenoid. We present the design, actuation principle and fabrication of EuMoBot, a soft robot demonstrating euglenoid movement. By novel application of quantitative shape descriptors, its locomotion through a fluid is quantified and compared to that of a euglenoid and similarities are discussed. However, due to the multi-modal nature of operation of the robot, it is not restricted to movement in a fluid and is demonstrated moving on a hard flat surface and within an empty pipe.

### 1.1. Euglenoid movement

During euglenoid movement, the cell exhibits a large change in its shape. These shapes range from a spherical form to an elongated rod-like form with a wide range of intermediate shapes [3]. Shown in figure 2 are photographs of *Eutreptiella spirogyra* from The Euglenoid Project [8] as it performs the characteristic locomotion. The progression of shapes during one cycle of locomotion can be described as a wave of expansion and contraction passing over the entire length of the body, similar to peristalsis. This wave starts from the anterior of the cell and propagates to the posterior, resulting in a forward motion of the body.

The changes in shape of the cell during euglenoid movement have been studied extensively. Measurements along the length of the cell in *Euglena fusca* were taken in [4] and the shape was approximated using a mathematical function. The cells studied were approximately 160 µm long. Analysis revealed a contraction in length of about 37% in the longitudinal direction and a doubling of radius during rounding up of the cell. A more detailed analysis of the shape including methods of modelling is presented in [5].

The underlying mechanism that causes changes in shape has been studied in [3]. The surface of a euglenoid cell is covered in proteinaceous strips called pellicles [10]. Sliding of these strips against each other allows the cell to exhibit its wide variety of shapes. Actuation at the microscopic scale of the pellicles causes a macroscopic change in the shape of the cell. This concept has been described mathematically in [6] and a method to generate euglenoid-like shapes has been presented. In this paper, we focus on realizing the macroscopic shapes of the euglenoid in a robotic form, without replicating the changes occurring at the microscopic level, such as pellicular actuation.

### 1.2. Soft actuator technologies

Several attempts have been made to design worm-like robots using soft actuator technologies. A review of relevant designs that enable large changes in body shape are presented here. Dynamic mesh-like structures have been employed in the SoftWorm [11] and the CMMWorm [12]. However, these require large structures to accommodate rigid actuators and are restricted in expansion. The Meshworm [13] in contrast, is completely soft and flexible. Coiled shape memory alloy actuators [14] are used to achieve high strain. However, these actuators have a slow actuation–relaxation cycle. Tendon-driven soft robots [15] that use change in shape to crawl-like caterpillars have also been developed. The change in shape in these robots helps in generating asymmetry in friction that aids the robot move itself. Granular jamming [16] is another completely soft approach and has been applied in [17] to realize a robot that moves by changing stiffness of various sections. Despite its ease of fabrication, it is not clear if actuation of this type can produce the desired amount of strain observed during deformations of euglenoids.

**Figure 3.**
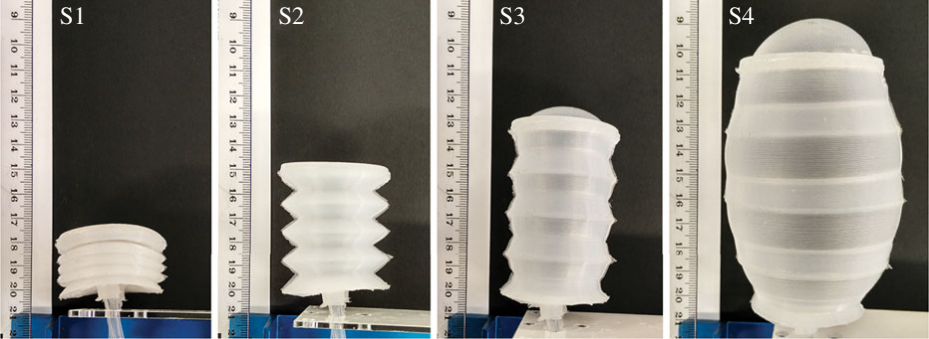
States of actuation of the hyper-elastic bellows actuator [7]: minimal length axially compressed state (S1), rest bellows state (S2), axially elongated state (S3) and ballooned state (S4). Copyright © IEEE, 2017. (Online version in colour.)

Soft fluidic actuators exhibit rapid expansion [18], require minimal control and show repeatable actuation–relaxation cycles. Exploiting these advantages, we have designed a novel bellows-like device termed the hyper-elastic bellows (HEB) actuator. The design and characterization of the actuator is presented in [7]. This device captures and separates the two key features of euglenoid movement: axial extension and contraction, and radial expansion. The folded bellows-like structure allows for operation under both positive and negative pressures causing axial expansion and contraction, respectively, while the hyper-elastic nature of the material allows for unconstrained expansion in radius. This is unlike the designs presented in [19,20] where the pneumatic chambers are constrained by design. Figure 3 shows the principle of operation of the actuator along with the four stages of actuation. Also discussed in [7] are methods of optimizing the design of the HEB for expansion using finite-element analysis. A comparison with other classes of fluidic actuators is also presented.

**Figure 4.**
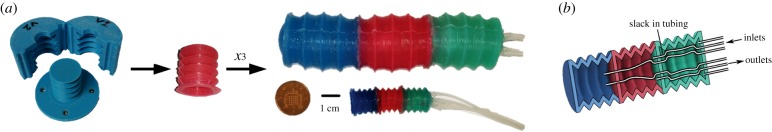
(*a*) Fabrication of the soft robot. Left: three-dimensional printed mould with two halves and a core used to cast the silicone elastomer. Middle: a single actuator chamber. Right: three-segment robot fabricated at two different scales. An English penny is shown for comparison. (*b*) Cross-sectional view of the robot showing the different chambers. Each chamber was supplied with an inlet and an outlet tube. Sufficient slack was provided in the tubes to allow for free expansion of the chambers. (Online version in colour.)

The multi-segment soft robot presented in this paper has been fabricated by bonding together three HEB actuators (figure 4). We will subsequently analyse the locomotion of this robot and compare its behaviour with that of the euglenoid.

## 2. Methods

### 2.1. Fabrication

Each HEB actuator unit was cast separately out of two-part silicone elastomer (Dragon Skin 10 SLOW, Smooth-On). A three-dimensional printed mould was used to form the shape of the actuator. It consists of a central core and two outer halves enclosing it (figure 4*a*, left). Uncured elastomer was injected into this mould and left to cure overnight. Three actuator chambers were cast separately and bonded to each other using an adhesive (Sil-Poxy, Smooth-On). The chambers were isolated from each other by a layer of silicone, 1 mm in thickness. Two robots were fabricated at different sizes to evaluate the effect of scale. The larger robot has a total length of 110 mm and a diameter of 30 mm, while the smaller one is 45 mm long and has a diameter of 12 mm.

The elastomer cures to a translucent white colour. To visually distinguish the chambers, pigments (Silc-Pig, Smooth-On) were mixed with the uncured elastomer before casting. Two thin silicone tubes with an inner diameter of 0.8 mm and outer diameter of 1 mm were fed through to each chamber and bonded in place. Sufficient slack was provided to allow for free expansion of the actuators. A cross-sectional schematic of the robot is shown in figure 4*b*.

Design of the HEB actuator, such as the angle of the folds, affects its behaviour. In previous work [7], a finite-element analysis of the expansion in axial direction for different fold angles was performed, which showed that increasing the angle of folds reduced the pressure required to achieve full ballooning of the actuator. Additionally, the behaviour was compared to that of other commonly used soft actuators such as rigid bellows and McKibben air muscles. The effect of parameters such as shape of the folds [21] and their density on axial and radial expansion was discussed. Here we adopt the same design parameter (half angle of 38.66°). Analysis of change in principal components for three designs of an HEB actuator module, including their relation to actuation parameters is presented in the electronic supplementary material.

### 2.2. Experimental set-up

To actuate the robot, a fluid pump with a flow rate of 250 ml s^−1^ was used to move fluid in and out of each chamber. To reproduce a suitably low Reynolds number regime (estimated *Re* = 10^−4^ for euglenoid swimming [5]), a solution of methyl cellulose (M0512, Sigma-Aldrich) in water is used for the robot to swim through. At a concentration of 1% w/w, this solution has a viscosity of 1000 cP. The approach of using a more viscous medium when working with larger scale experimental models has been shown to give insights into the dynamics of smaller physical systems such as insect wings [22]. This is the reason for the choice of the experimental medium which replicates the Reynolds number at the length and velocity scale of the robot. Scaling of physical properties in biological systems with the size and shape of organisms is also discussed in [23]. Water is used as the fluid of operation inside the robot to make it neutrally buoyant in the viscous medium.

**Figure 5.**
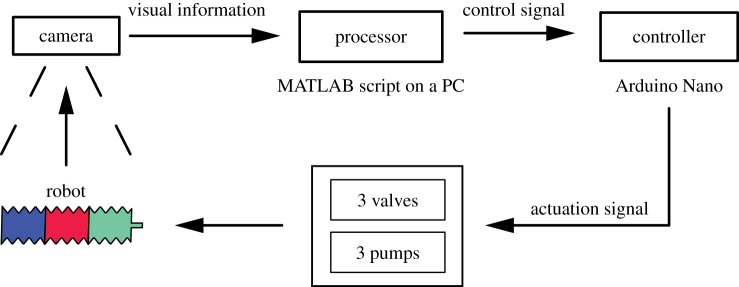
Schematic view of the control architecture of the robot. A camera is used to determine the current state of the robot. A MATLAB script processes that information to determine the current state of the robot, i.e. the expansion and contraction of each chamber. It then passes appropriate control signals to an Arduino which controls the operation of pumps and valves to direct the flow of fluid inside the robot. (Online version in colour.)

A feedback-based approach was used to autonomously control the robot (figure 5). This removes the need to model the dynamics of the pump and the flow of fluid within the robot. Feedback to the controller was provided through a machine-vision estimate of chamber size, using a camera (Microsoft LifeCam HD-3000, 30 fps) and a MATLAB script. Each chamber was cast in a separate colour, blue, red or green, and the size of the chamber was estimated in terms of the number of pixels of that colour in the image. The state of expansion and contraction of each chamber of the robot is thus obtained and used to control the flow of fluid inside the robot by turning the corresponding pumps on or off. Initial experiments incorporated sensors to measure the pressure inside each chamber. The sensors were located outside the robot and directly measured the pressure of fluid in the tubes carrying fluid into the chamber. However, frictional pressure loss due to the thin diameter of tubes and time-related dynamics of the pump resulted in inaccurate measurement of internal chamber pressures. The machine-vision method proved to be more consistent and did not require alterations to the robot other than in its colour.

### 2.3. Shape estimation

The robot takes a range of shapes during its locomotion. The motivation to formally characterize these shapes is twofold. One is to understand how the robot changes shape during locomotion. The other is to quantitatively compare the shape of the robot and the organism. This requires the use of a mathematical descriptor of the shape.

Since the shapes of the organism and the robot have been extracted from video recordings and the viewpoint might change between recordings, it is desired that the chosen descriptor be invariant to translation, rotation and scale. The elliptic Fourier descriptor for closed contours, presented in [24] has been chosen because it satisfies the required criteria. It is a procedure to fit a closed curve to a set of two-dimensional points with arbitrary precision. This descriptor has been extensively used to describe biological shapes such as those of nuclei [25], shells [26], leaflets [27] and roots [28]. The novelty in applying it in this work is in describing the shape of a nonlinear, dynamic, hyper-elastic soft robot and in comparing it with that of a biological organism. The work in [5] employed a complementary approach, where the cell was assumed to be axisymmetric and a B-spline cure was fit to one half of the shape. The elliptic Fourier descriptor is more general and does not assume such a condition.

The elliptic Fourier descriptor is extracted in four main steps. First, individual image frames are extracted from video and processed to improve the contrast of the desired object from the background. In the case of euglenoid, manual processing of frames was necessary because the organism appears transparent under certain lighting conditions. In the case of the robot, tuning by hand was not necessary as the video was recorded against a contrasting background at a constant focal length and frame rate. The region of interest was obtained by applying a suitable threshold in the HSV colour space.

In the second step, the contour of the object is extracted. A 3 × 3 kernel median filter is used on the pre-processed image to remove noise and any holes in the region of interest are filled. When the image is left with only one identified region of interest, the boundary of this region is extracted. The contour is a set of pixel coordinates that is a discrete representation of the boundary.

Next, the coordinate encoding the boundary is transformed to a chain of integers as described by Freeman [29], resulting in a piece-wise linear approximation of the contour which contains information of the local orientation of the curve.

**Figure 6.**
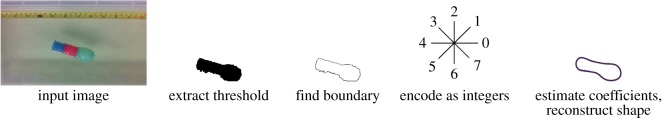
The four stages of shape estimation. First the region of interest is extracted from the image. Next, the boundary is identified. This contour is then encoded as a set of Freeman integers. Finally, a set of Fourier coefficients is calculated and the shape is reconstructed. (Online version in colour.)

The last step consists of computing the Fourier coefficients of this encoding up to an arbitrary number of harmonics. These are a set of four coefficients per harmonic, two each describing the *x* and *y* projections of the contour. The shape of the object can then be reconstructed using a subset of these coefficients. The four steps of estimating the shape are shown in figure 6.

**Figure 7.**
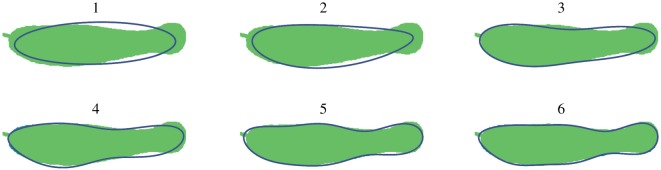
Estimate of the euglenoid cell shape as the number of harmonics increases from 1 to 6. Shaded region is the true shape. The outline in blue indicates the estimated shape. (Online version in colour.)

To determine the minimum number of harmonics needed to describe the shape, change in area of the estimated shape was used as the determining measure. Beyond a certain number of harmonics, the area enclosed by the approximated curve does not change significantly. This number was taken to represent the curve with sufficient detail and is a representation of the complexity of the shape. Figure 7 shows this pictorially in the case of the euglenoid. Five harmonics were found to be sufficient for the shape of the euglenoid and six harmonics were used for those of the robots.

### 2.4. Shape characterization

The set of Fourier coefficients obtained as described above, constitute the mathematical description of the shape. The coefficients can be normalized to make the descriptor invariant to rotation, translation and scale [16]. An approximation using *N* harmonics, results in a descriptor with 4*N* – 3 normalized coefficients. For a motion sequence consisting of *M* frames, the description of the motion is then a matrix of *M* × 4*N* − 3 coefficients. These coefficients themselves have little physical meaning. The goal then is to extract key descriptors that describe the shape in a physical sense. This is achieved through reduction of the coefficients into principal components, along the lines of [30]. Locomotion in the low Reynolds number regime being considered here is driven by change in shape and the principal components that capture features of these shapes are thus also descriptors of locomotion.

The amount of variance in shape captured by an eigenvector is used as a quantitative measure of similarity in shape between the robot and the organism. Let **D_e_** and **D_r_** represent matrices of Fourier coefficients of the euglenoid and the robot, respectively, where each row corresponds to one shape. Let **X_e_** and **X_r_** represent matrices whose columns contain the eigenvectors of the covariance matrix of **D_e_** and **D_r_**. The variances of the columns in the product matrices **D_e_X_e_** and **D_r_X_r_** are the corresponding eigenvalues. These represent the amount of variance in shape explained by each eigenvector. The variances of columns in the product matrices **D_e_X_r_** and **D_r_X_e_** represent the ability of the eigenvectors of one sample to explain the variation in shape in the other sample. This set of eigenvalues computed between samples is thus a quantitative measure of similarity.

## 3. Results

### 3.1. Replicating euglenoid shapes

**Figure 8.**
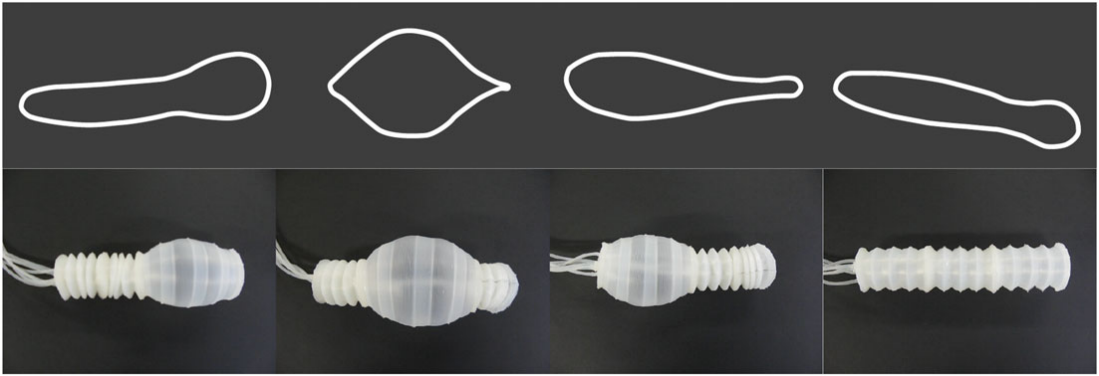
Top: outlines of cell shapes from figure 2 during euglenoid movement. Bottom: the soft robot replicating euglenoid shapes. (Online version in colour.)

Inflating and deflating chambers of the robot enables it to replicate changes in shape displayed by the euglenoid. Shown in figure 8 is a comparison between the robot and outlines of the cell from figure 2 demonstrating similarity of achievable shapes.

### 3.2. Robot locomotion

To test the locomotion ability of the robots, each of them was placed in a tank filled with a solution of methyl cellulose. Fluid inside the robot was moved from one chamber to the next, starting from the anterior end and moving towards the posterior, reproducing the motion of an expansion wave as is the case in euglenoids. When the last chamber expanded fully the cycle was reset by moving the fluid directly to the foremost chamber, skipping all intermediate chambers (figure 10*b*). The internal area of the chamber is plotted for one of the trials in figure 10*a*. Euglenoids have been observed to both change in volume and maintain a fixed volume during euglenoid movement. Exact values of volume change are not readily available due to the wide variety of observed shapes, but one study reported a 20% change in volume in one of the many cells studied [5]. The robots in all experiments were operated with constant internal volume. They do however, possess the ability to operate with variable volume if desired. At the beginning of the cycle, the foremost chamber was inflated to a volume six times that of its resting state, while the other chambers remained contracted.

**Figure 9.**
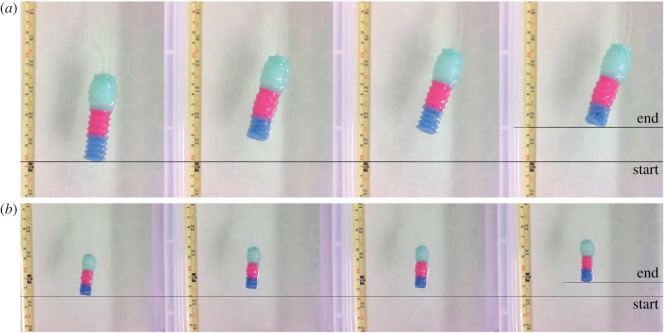
A sequence of three cycles of locomotion of the robot; (*a*) larger version of the robot and (*b*) the smaller version. (Online version in colour.)

**Figure 10.**
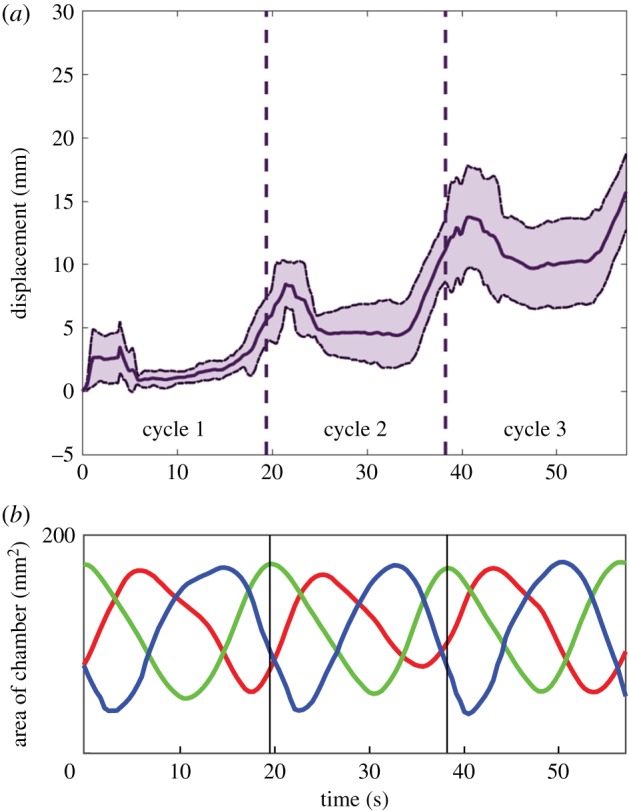
(*a*) Displacement of the centroid of the smaller robot during three cycles of motion. The solid line represents mean of three trials and the dotted line is one standard deviation away from the mean. (*b*) The change in area of individual chambers (smoothed with a moving average filter with window span of 15 points) during one of the trials is shown below, each curve corresponding to the chamber of the same colour. (Online version in colour.)

**Figure 11.**
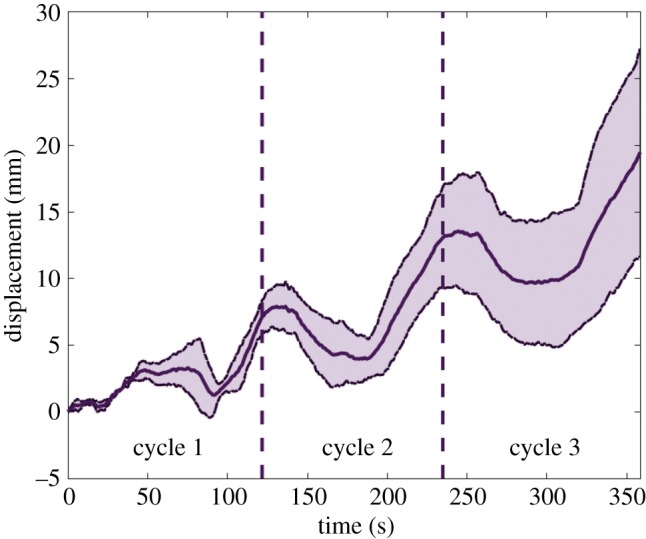
Displacement of the centroid of the larger robot during three cycles of motion. The solid line represents mean of three trials and the dotted line is one standard deviation away from the mean. (Online version in colour.)

Figure 9 shows a sequence of images captured during the motion of the two robots (electronic supplementary material, video S1). The centroid of the robot was tracked during locomotion. Figures 10 and 11 show the mean and standard deviation in the displacement of the centroid during three steps of locomotion. The averages were computed across three trials with each robot. The smaller robot moved at an average velocity of 20 mm min^−1^ (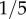 body lengths per cycle, 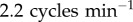). In the case of the larger robot, the average velocity was 4.5 mm min^−1^ (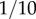 body lengths per cycle, 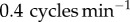). This is comparable to the estimated speed of swimming in a euglenoid, which is also 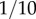 body lengths per cycle [5].

### 3.3. Shape change during euglenoid movement

**Figure 12.**
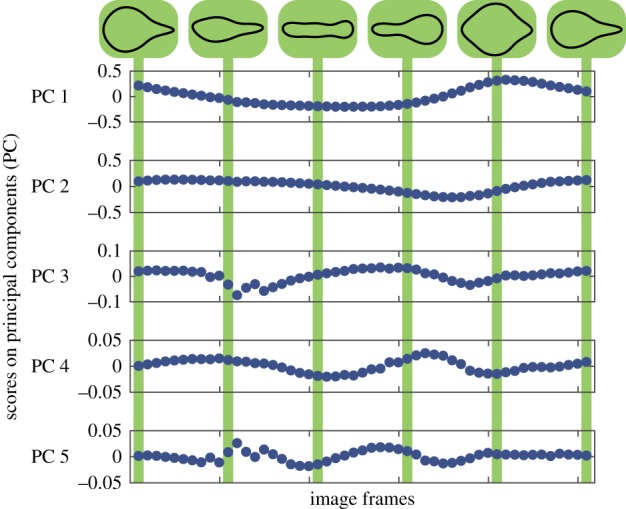
Change in scores on principal components describing the shape of the organism during one cycle of euglenoid movement. Reconstructed shapes are shown above for the scores indicated in the plots. (Online version in colour.)

Video of the euglenoid from [8] was analysed and the outline of the cell was extracted from each frame. Shape was estimated using the elliptic Fourier descriptor as described in §2.3. The estimated Fourier coefficients were reduced to a set of five principal components to capture the variation in shape across all frames. Figure 12 shows the change in scores on these five principal components during one cycle of the motion of the euglenoid.

**Figure 13.**
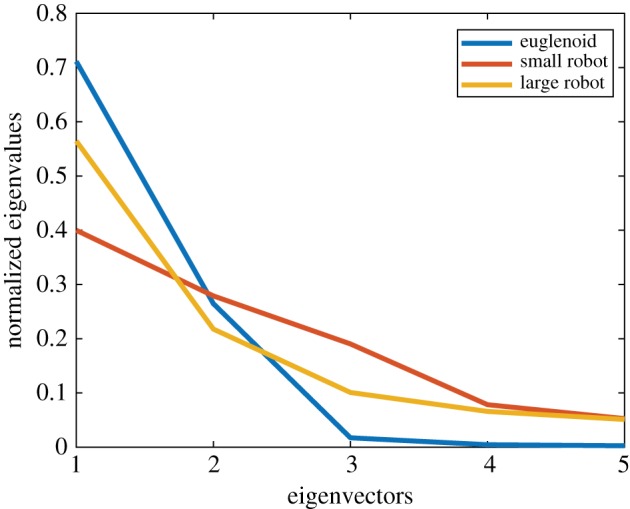
Normalized eigenvalues of the first five eigenvectors describing the shape. (Online version in colour.)

**Figure 14.**
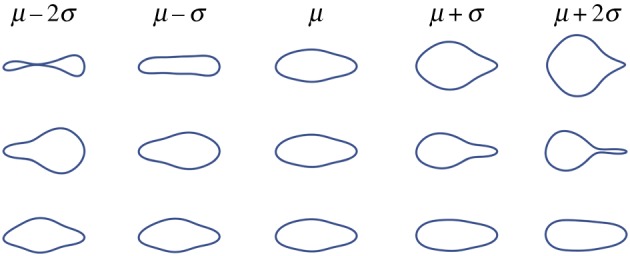
Effect of change in scores of each principal component on the estimated shape of the euglenoid. Each row corresponds to change in score of one principal component. The central column represents the mean shape. On either side are one and two units of standard deviation away from the mean. (Online version in colour.)

Figure 13 shows the eigenvalues corresponding to the first five eigenvectors. In the case of the euglenoid, it is clear that the first three eigenvectors capture most of the variation in shape, with a cumulative variance of 99.3%. To illustrate the effect that each principal component has on the shape, the scores on each component were varied independently while those on the other components were fixed at their means. The effect of these changes is shown in figure 14 for the first three principal components derived from the euglenoid video. The central column shows the shape of the organism when scores on all the principal components are set to their means. Each row shows the effect of change in one principle component. Each column away from the centre represents a change of one standard deviation.

The first principal component has a major contribution towards the width of the cell, thereby affecting the rounding up of the cell. The second component captures the shifting of mass between the anterior and posterior of the organism. The third component captures width of the central portion of the cell.

### 3.4. Shape change during locomotion of the robot

**Figure 15.**
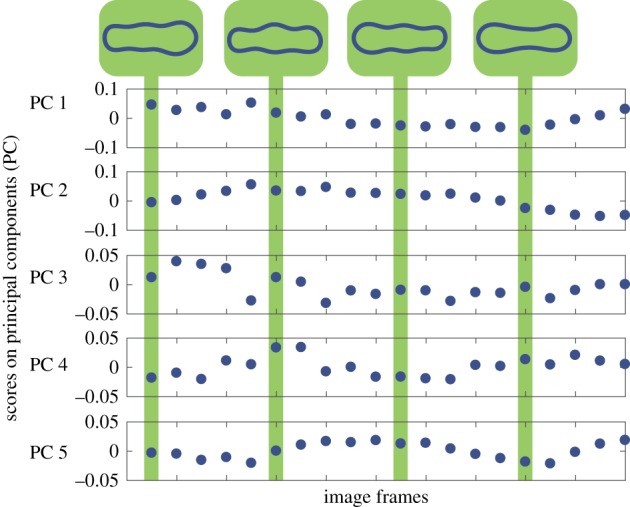
Change in scores on principal components describing the shape of the smaller robot during three cycles of locomotion. Reconstructed shapes are shown above for the scores indicated in the plots. (Online version in colour.)

**Figure 16.**
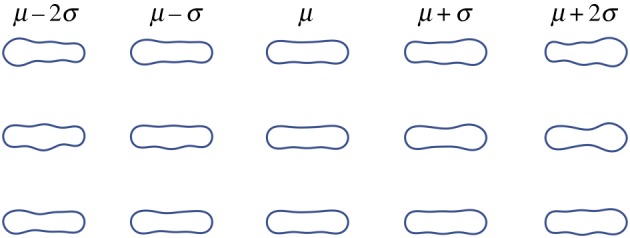
Effect of change in scores of each principal component on the estimated shape of the smaller robot. Each row corresponds to change in score of one principal component. The central column represents the mean shape. On either side, are one and two units of standard deviation away from the mean. (Online version in colour.)

Videos of the robot in locomotion were analysed in an analogous manner. Scores on the first five principal components are shown in figure 15 for the smaller robot. The eigenvalues are shown in figure 13. In the case of the smaller robot, the first three eigenvectors capture a cumulative variance of 86.9% and in the case of the larger robot, 88.3%. Figure 16 shows the change in shape of smaller robot as the scores on the principal components are varied. The corresponding figures for the case of the larger robot can be found in the electronic supplementary material.

In both the robots, the first principal component describes the position of the widest region of the robot. The second component in case of the smaller robot and the third component for the larger robot describe an hourglass shape as the score increases and its inversion when the score decreases. The other component describes a subtler feature of the shape.

### 3.5. Comparison of shapes

The method described in §2.4 was used to compare the shapes. Videos of one cycle of locomotion were considered and sampled to have the same number of frames. Six harmonics were used to estimate all shapes. Fourier coefficients were reduced to three principal components. Using coefficients from one sample and eigenvectors from another sample, the variance of the first three principal components has been computed (table 1). The higher the variance, the better the ability to capture details of shape. It can be seen that the first principal component of the large robot, which corresponds to anterior–posterior mass transfer, captures 78.78% of the variance in euglenoid cell shape. The second principal component of the euglenoid, again corresponding to shifting of mass, captures 84.95% of the variance in shape in the larger robot. The corresponding numbers for the smaller robot and the euglenoid are 30.33% and 55.19%.

**Table 1.** Variance in shape captured by principal components.

coefficients [*D*]	eigenvectors [*X*]	normalized variance of first three principal components [*DX*]
euglenoid	large robot	78.78%, 5.96%, 15.26%
large robot	euglenoid	9.3%, 84.95%, 5.75%
euglenoid	small robot	30.33%, 14.33%, 5.53%
small robot	euglenoid	28.74%, 55.19%, 24.26%

**Figure 17.**
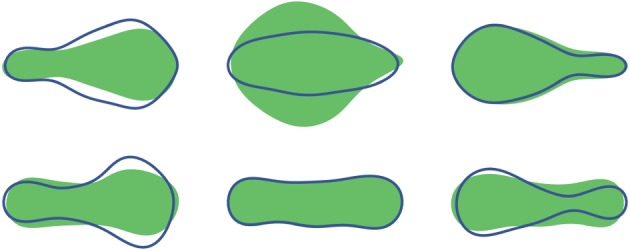
Comparison of shapes between euglenoid and the larger robot at three different instances during one cycle of locomotion. Top row: shaded region indicates the true shape of the euglenoid. The outline in blue is the shape estimated using the scores on the first principal component from the shape of the larger robot. Bottom row: shaded region indicates the true shape of the larger robot. The outline in blue is the shape estimated using the scores on the second principal component from the shape of the euglenoid. (Online version in colour.)

**Figure 18.**
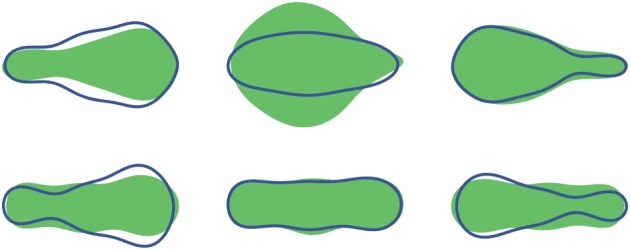
Comparison of shapes between euglenoid and the smaller robot at three different instances during one cycle of locomotion. Top row: shaded region indicates the true shape of the euglenoid. The outline in blue is the shape estimated using the scores on the first principal component from the shape of the smaller robot. Bottom row: shaded region indicates the true shape of the smaller robot. The outline in blue is the shape estimated using the scores on the second principal component from the shape of the euglenoid. (Online version in colour.)

Based on these values, shapes of the euglenoid were reconstructed using the first principal component describing robot shapes. Similarly, shapes of the robot were reconstructed using the second principal component describing euglenoid shapes. These are shown in figures 17 and 18.

## 4. Discussion

### 4.1. Locomotion ability

Both the robots demonstrated the ability to swim by changing the shape of the body (figure 9). The disparity in speed of the larger robot could be attributed to the volume of fluid being pumped between chambers of the robot. A pump with a larger flow rate could improve the average velocity. The characteristic lengths for the smaller and larger robots are of the order of 10^−3^ m and 10^−2^ m, respectively. Their velocities are of the order of 10^−4^ m s^−1^ and 10^−5^ m s^−1^, respectively. Viscosity of the medium is 1 Pa s. The resulting Reynolds numbers are of the order of 10^−4^ for both the sizes, which represents the flow regime of the euglenoids.

As explained in the Introduction, the euglenoid needs to develop a force against the water to propel itself forwards. The mass of the euglenoid is so negligibly small that if the force of propulsion vanishes, it is estimated that the drag due to water stops the motion of the cell within 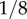 of its body length [31]. Therefore, the euglenoid maintains a series of propulsions generated by cyclic changes in body shape. Additional aspects of the dynamic interaction between the body and the environment such as the motion that generates the most thrust and the flow of fluid around the body have not been studied. Having shown the hydrodynamic similarity between the robot and the organism in terms of the Reynolds numbers at both the sizes, the robots can now be used as a test platform to elicit new insights into euglenoid movement.

**Figure 19.**
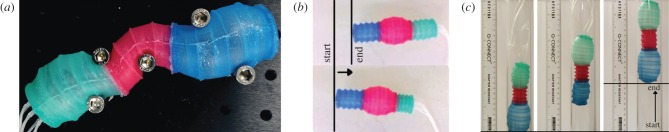
(*a*) Demonstration of compliance of the robot as it expands between rigid screws. (*b*) Sequence of images at the start and end of one cycle of locomotion of the robot on a flat table. (*c*) Robot climbing inside a pipe using an inch-worm type gait. (Online version in colour.)

Unlike the organism, the robot is not restricted to working in a fluidic environment and in addition to replicating euglenoid movement, the soft robot presented here is capable of multi-modal locomotion. Figure 19*b* shows the robot moving on a flat table and figure 19*c* shows the robot climbing a pipe with an inchworm gait, using a non-constant volume of operation (electronic supplementary material, video S2). Therefore, the robot can also be used to test and compare different locomotion strategies.

### 4.2. Similarity in shape

A key feature of euglenoid movement is the transport of internal mass from one end of the body to the other. This is observed in the diversity of shapes. The comparative analysis of shapes shows that the principal components capture this variation to a high degree and that the robots achieve these drastic changes in shape. Based on analysis of shape alone, this observation suggests that the robots will be able to achieve euglenoid-like locomotion given a suitable medium, which has already been proven in §3.2. That this conclusion could be drawn without the need for a model of euglenoid movement, demonstrates the mathematical elegance of the method used.

From the plot of eigenvalues in figure 13, it can be seen that the shape of the euglenoid is distinctly characterized by the first three principal components, whereas in the case of the robots, the variance is spread across more components. This might suggest that extreme shapes as seen in the euglenoids are not fully reproduced in the robots. The reconstructed shapes in figures 17 and 18 support this claim. On the other hand, based on the similarity in locomotion, it can be argued that such extreme shapes might not be necessary for propulsion as long as a sufficiently large wave of expansion travels along the length of the body. In the case of the euglenoids, efficiency of swimming using euglenoid movement is estimated to be around 1% [5]. Efficiency of the robots has not been investigated here but it might be possible to move with higher efficiencies with less extreme changes in shape which shall be taken up in future work.

An interesting question to answer would be the reason for the existence of extreme shapes in euglenoids and whether the organism actively tries to attain such a shape or if it is the result of constraints on its construction. The approach of using a robot in studying the effect of changes in body shape is appropriate to address this question because of the ease of generating shapes in a controllable manner. In this context, a quantitative method of comparing shapes is particularly useful.

A second difference between the robots and the organism is that transition of fluid from one chamber to another in the robot does not produce a smooth transition in the shape of the contour. The boundary wall that separates two chambers, though elastic, locally restricts the expansion of the chambers to a certain extent. This difference partially explains the inability of the robot to assume more extreme shapes. Given a sufficiently large number of chambers, the transition in shape could be made smoother. Based on eigenvalues of figure 13, and the variances shown in table 1, it can be seen that the larger robot reproduces shapes closer to the euglenoid than the smaller one. A possible reason for this difference could be the design of the folds of the bellows. The difference in shape between extremes due to expansion and contraction of folds is less pronounced at smaller scales. Parameters such as angle of folds, their number and density could be optimized to achieve better replication of shape.

## 5. Conclusion and future work

This paper describes the fabrication and analysis of locomotion of a multi-segment soft robot, EuMoBot, that replicates euglenoid movement. In addition to swimming with shape change, the robot is capable of locomotion in non-fluid environments. The soft and compliant nature of the robot (figure 19*a*) could be exploited for operating in constrained spaces. Future work on the robot will investigate efficiency of locomotion and changes in design and control required to improve its performance. The effect of parameters such as the frequency, amplitude and phase of actuating pressures in individual chambers on the locomotion will be studied. It is not clear as to what information about locomotion all the principal components provide. Components describing mass transport provide insights but others describing the roundness of shape do not. Further work is necessary to understand this relationship between principal components and locomotion ability.

The extremity of shapes exhibited by the robot is limited by the ultimate tensile strength of the material used for fabrication. Even though each chamber of the robot is unconstrained during operation, as the internal pressure increases, the actuator can fail due to rupture of the membrane. This limitation is primarily due to the monolithic skin design. Alternative fabrication techniques such as three-dimensional printing and soft lithography [32] could partially address limitations of the elasticity of the material. On the other hand, euglenoids do not suffer from this limitation, because they employ a different mechanism of achieving change in shape, namely sliding of pellicles. Unlike the robot, between two extreme shapes, there is negligible strain in the pellicle strips as they do not change in length but only slide against each other. This allows the euglenoid to transition between shapes without rupturing its cell membrane. Implementing the mechanism of pellicles in a robot is being considered and is a topic of future research. This demonstrates a fundamental challenge in achieving giant shape change in robotics; to exceed the mechanical limits of the material requires multi-element structures with moving parts.

Another limitation of the current design is the inability of the robot to change direction. Ability to steer could be added to the current design by adding mechanisms to orient chambers in the desired direction. Using actuated coils of shape memory alloy [33] or using constraints on the material [19] are two of several possible solutions. Sources of power, actuation and control could be placed within the body of the robot [34], eliminating the need for a tether and thus reducing the size of the robot.

The quantitative method of comparison based on elliptic Fourier descriptors was used to show similarity in shapes of the organism and the robot. The method of comparison presented here could potentially be used for other soft robotic systems where accurate models of shape do not exist. Hydrodynamic similarity between the robot and the organism was established. The robot can therefore be used as a tool to study the dynamics of locomotion in euglenoids and possibly other organisms, thereby contributing to the understanding of biology.

## Supplementary Material

EuMoBot: Replicating Euglenoid Movement in a Soft Robot (Supplementary Material)

## Data accessibility

Data presented in this study are available from the University of Bristol’s research database and can be accessed using the doi: 10.5523/bris.1ymwu1wak9x4g2rowm1ogfrjze.
