# Supplementary material for: EuMoBot: replicating euglenoid movement in a soft robot
Source: J R Soc Interface. 2018 Nov 21;15(148):20180301. doi: 10.1098/rsif.2018.0301 (PMC6283989; doi:10.1098/rsif.2018.0301)
Supplement: EuMoBot: Replicating Euglenoid Movement in a Soft Robot (Supplementary Material) [file rsif20180301supp1.pdf]

EuMoBot: Replicating Euglenoid Movement in a Soft Robot (Supplementary Material) Journal of the Royal Society Interface

Krishna Manaswi Digumarti[1], Andrew Conn[1,2], and Jonathan Rossiter[1,3]

[1]*Bristol Robotics Laboratory, University of Bristol and University of the West of England, BS16 1QY, UK. (email: km.digumarti@bristol.ac.uk)*
[2]*Department of Mechanical Engineering, University of Bristol, BS8 1TR, UK*
[3]*Department of Engineering Mathematics, University of Bristol, BS8 1UB, UK*

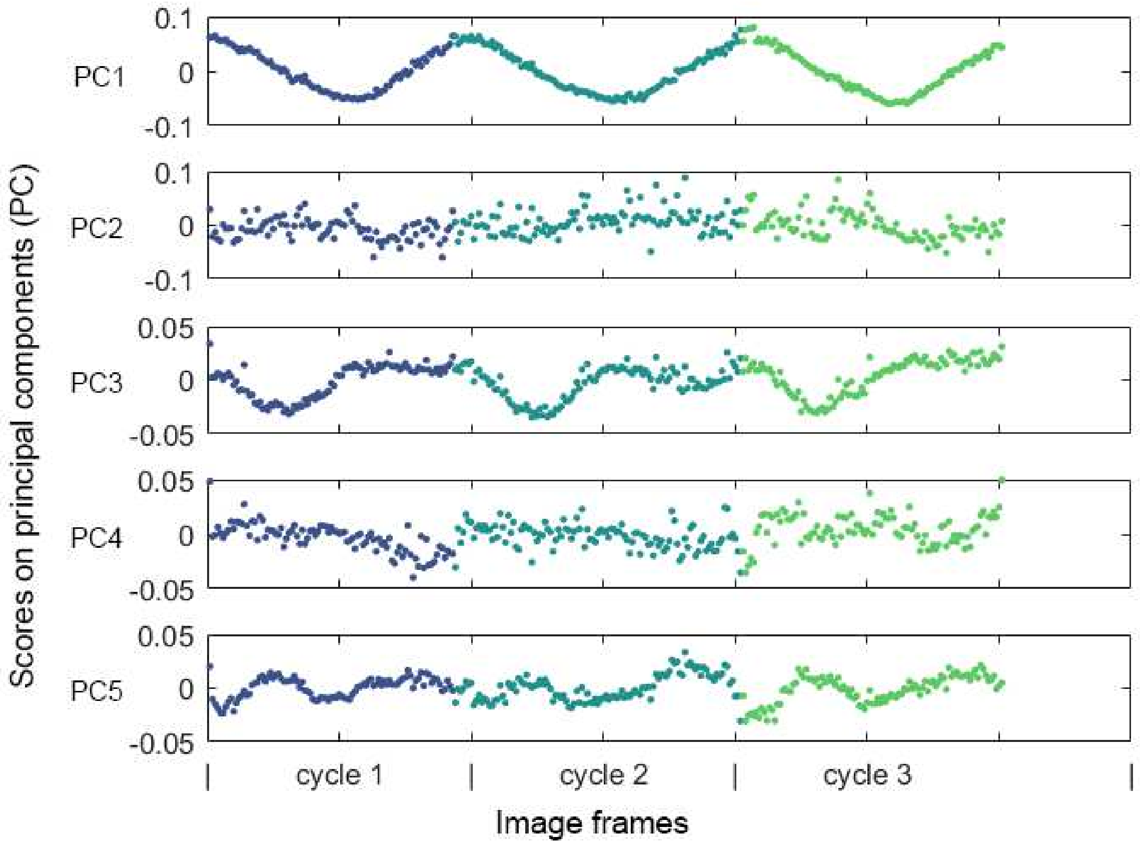

Figure 1: Change in scores on principal components describing the shape of the larger robot during three cycles of locomotion.

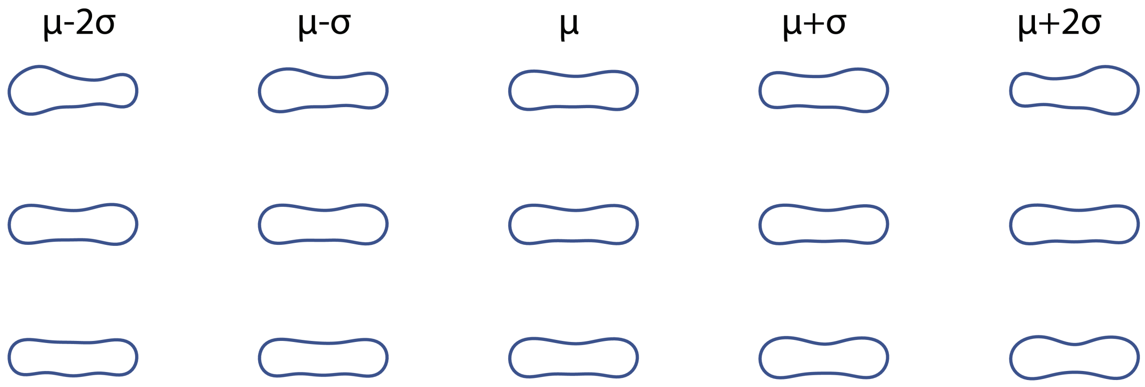

Figure 2: Effect of change in scores of each principal component on the estimated shape of the larger robot. Each row corresponds to change in score of one principal component. The central column represents the mean shape. On either side, are one and two units of standard deviation away from the mean.

PCA for different design parameters

In this section, we consider three different designs of the HEB actuators used in the construction of the robot. As presented in [1], the behaviour of the actuator can be characterised in terms of the relation between diameter and length with internal pressure. We use a piecewise linear approximation of the behaviour and consider three designs for the actuator unit which vary in terms of the steepness of the change in diameter as the length of the actuator increases (fig. 3a). The curves make an angle of 30°, 45° and 60° with the horizontal respectively. Initial length of the actuator was taken as 15mm and minimum diameter as 12mm, which is the case with those used in the smaller robot. Upon actuation, is was assumed that the length linearly increases from 15mm to 30mm. This is a reasonable assumption as a doubling in length was observed in the experiments. The maximum diameter was then determined using the relation shown in the figure. The shape of the actuator was approximated by a second order curve, abstracting away the folds of the bellows as shown in fig. 4.

For each of the three designs, 16 shapes were constructed during the expansion phase (length increases from 15mm to 30mm). The shapes were then represented using the elliptic Fourier descriptor and analysed using PCA as described in the paper. The first two principal components are shown in fig. 3b, 3c. The first component describes the change in length of the actuator and the second component describes its roundedness.

In addition to showing how the components change with design parameters, this analysis also gives insights into the relation between actuation parameters (amplitude and frequency) and the principal components. The plots in fig. 3b, 3c correspond to one half of each actuation cycle, with the other half being the contraction phase of the actuator. Multiple cycles of actuation can thus be represented by repeating the curves several times, after appropriate inversion for the contraction phase. Amplitude of actuation is represented in this analysis as a single degree of freedom in terms of the actuator length. Change in shapes for a smaller amplitude can thus be represented by restricting the range of length and using parts of the curve. A change in frequency of actuation does not alter the shape of the actuator but only alters the rate at which the same shape is observed.

[1]K. M. Digumarti, A. T. Conn, and J. Rossiter, Euglenoid-inspired giant shape change for highly deformable soft robots, IEEE Robotics and Automation Letters, vol. 2, no. 4, pp. 23022307, 2017.

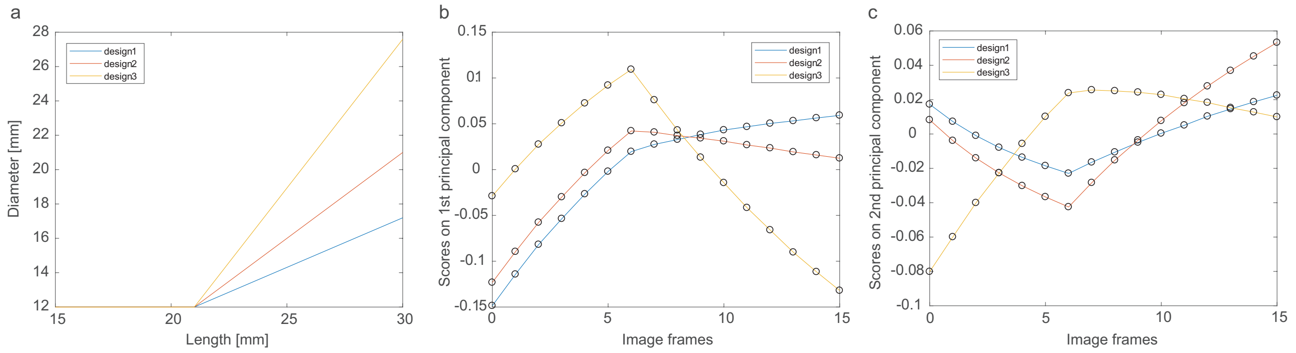

Figure 3: (a) Diameter of the actuator as a function of its length for three different designs The rising parts of the curve make an angle of 30°, 45° and 60° with the horizontal respectively. (b) Scores on the first principal component for shapes of the actuator during its expansion phase. (c) Scores on the second principal component for shapes of the actuator during its expansion phase.

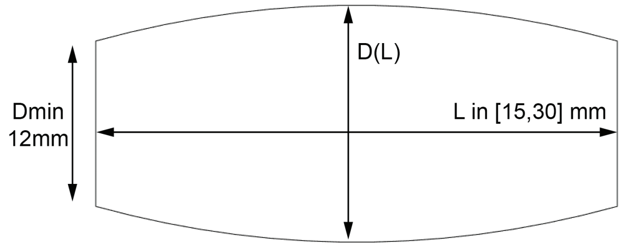

Figure 4: Approximated shape of the HEB actuator as length changes from 15mm to 30mm.
